# Local administration of glucocorticoids decreases synovial citrullination in rheumatoid arthritis

**DOI:** 10.1186/ar3702

**Published:** 2012-01-27

**Authors:** Dimitrios Makrygiannakis, Shankar Revu, Marianne Engström, Erik af Klint, Anthony P Nicholas, Ger JM Pruijn, Anca I Catrina

**Affiliations:** 1 Department of Medicine, Rheumatology Unit, Karolinska University Hospital, Karolinska Institutet, SE- 141 86, Stockholm, Sweden; 2 University of Alabama at Birmingham and Birmingham Veterans Administration Medical Center, S. 19th Street, Birmingham, AL 35233, USA; 3 Department of Biomolecular Chemistry, Radboud University Nijmegen, PO Box 9102, 6500 HC, Nijmegen, The Netherlands

## Abstract

**Introduction:**

Protein citrullination is present in the rheumatoid synovium, presumably contributing to the perpetuation of chronic inflammation, in the presence of specific autoimmunity. As a result, the present study examined the possibility that effective antirheumatic treatment will decrease the level of synovial citrullination.

**Methods:**

Synovial biopsies were obtained from 11 rheumatoid arthritis (RA) patients before and after 8 weeks of treatment with 20 mg methotrexate weekly, 15 RA patients before and 2 weeks after an intraarticular glucocorticoid injection, and eight healthy volunteers. Synovial inflammation was assessed with double-blind semiquantitative analysis of lining thickness, cell infiltration, and vascularity by using a 4-point scale. Expression of citrullinated proteins (CPs) with the monoclonal antibody F95 and peptidylarginine deiminase (PAD) 2 and 4 was assessed immunohistochemically with double-blind semiquantitative analysis. *In vitro *synovial fluid (SF), peripheral blood (PB), mononuclear cells (MCs), and synovial explants obtained from RA patients were incubated with dexamethasone and analyzed with immunohistochemistry for expression of CP as well as PAD2 and PAD4 enzymes.

**Results:**

The presence of synovial CP was almost exclusive in RA compared with healthy synovium and correlated with the degree of local inflammation. Treatment with glucocorticoids but not methotrexate alters expression of synovial CP and PAD enzymes, in parallel with a decrease of synovial inflammation. *Ex vivo *and *in vitro *studies suggest also a direct effect of glucocorticoids on citrullination, as demonstrated by the decrease in the level of citrullination and PAD expression after incubation of SFMC and synovial explants with dexamethasone.

**Conclusion:**

Synovial citrullination and PAD expression are dependent on local inflammation and targeted by glucocorticoids.

## Introduction

Rheumatoid arthritis (RA) is a chronic inflammatory disease characterized by the presence of highly specific anti-citrullinated protein antibodies (ACPAs) [1]. These antibodies recognize several different proteins that are citrullinated. Citrullination is the conversion of peptidylarginine to peptidylcitrulline through a calcium-dependent process catalyzed by the peptidylarginine deiminase (PAD) enzymes. Five PAD isotypes have been described in humans (PAD1, PAD2, PAD3, PAD4, and PAD6), which are expressed in a variety of tissues, but only PAD2 and PAD4 have been found to be expressed in inflamed synovial tissue of RA and other inflammatory arthritides [2].

Despite the high specificity of ACPA in RA in comparison to other arthritides and other inflammatory diseases [3], the presence of CP is not restricted to RA synovial tissue [4,5], but rather associated with inflammation in general [6]. Synovial citrullination appears therefore not to be essential for the predisease phase of induction of specific anti-citrulline immunity. Conversely, protein citrullination enhances the HLA binding capacity of synovial-derived proteins and promotes NF-κB and tumor necrosis factor (TNF) production in the presence ACPA [7]. This suggests that local synovial citrullination might be essential in a later phase of the disease process, contributing to occurrence and perpetuation of chronic synovitis in the presence of specific anti-citrulline antibodies. It is therefore conceivable that downregulation of synovial citrullination by any means will contribute to the resolution of local inflammation. We hypothesize that effective antirheumatic treatment with either antiinflammatory, intraarticular glucocorticoids (GCs), or a disease-modifying antirheumatic drug, such as methotrexate (MTX) will decrease synovial citrullination *in vivo*. As a result, the present study aimed to investigate any direct effect of these drugs on protein citrullination.

## Materials and methods

### Patients

Twenty-six patients meeting the 1987 American College of Rheumatology criteria for RA [8] were recruited for this study. In a first group, 11 patients (six women and five men; median age, 56 years; range, 33 to 78 years) with newly diagnosed RA (symptom duration less than 1 year) previously disease-modifying antirheumatic drug (DMARD) naïve were started on MTX, 10 mg weekly, and reached a stable dose of 20 mg after 2 weeks, increasing the dose with 5 mg every week. Synovial biopsy samples were obtained by arthroscopy from all patients before and after a median of 8 weeks of treatment. Clinical evaluation of the therapeutic response according to EULAR response criteria was performed a median of 3 months after methotrexate initiation. In a second group, 15 RA patients (11 women and four men; median age, 63 years; range, 34 to 83 years) with active knee arthritis independent of disease duration received an intraarticular injection of 40 mg of triamcinolone hexacetonide, and synovial biopsy samples were obtained with arthroscopy before and after 2 weeks after intraarticular treatment. In this second group, associated DMARD treatments were stable for at least 2 weeks before initiation of treatment and throughout the entire study period. Clinical evaluation of the therapeutic response was performed with macroscopic scoring of the level of inflammation during arthroscopy. Nonsteroidal antiinflammatory drugs and per-oral prednisolone to a maximum dose of 10 mg daily were permitted in both groups. Additionally, synovial biopsy samples were obtained with arthroscopy in eight healthy volunteers. All procedures were approved by the Northern Stockholm Ethical Review Board, and informed consent was obtained from all the participants in the study.

### Synovial biopsies handling

Synovial biopsy samples were snap-frozen during arthroscopy in dry-ice cooled isopentane. Serial cryostat sections (7 μm) were fixed for 20 minutes with 2% (vol/vol) formaldehyde and stored at -70°C.

### Cell culturing

Paired peripheral blood mononuclear cells (PBMCs) and synovial fluid mononuclear cells (SFMCs) from RA patients (*n *= 6) were cultured as duplicates in RPMI 1640 supplemented with 20% heat-inactivated fetal bovine serum (FBS), 2 m*M *L-glutamine, 50 IU/ml penicillin, and 50 μg/ml streptomycin (Gibco, Carlsbad, CA, USA) and incubated at 37°C with 5% CO_2 _in a humidified atmosphere. Cells were grown at a density of 1 × 10^6 ^per ml in polypropylene tubes and incubated with dexamethasone for 24 hours at a final concentration of 1 and 100 μ*M *DXM. After treatment, cells were washed, resuspended in PBS, and seeded on glass slides (Thermo Scientific diagnostic microscopic glass slides, Braunschweig, Germany). Adherent cells were fixed for 20 minutes at 4°C with 2% (vol/vol) formaldehyde (Merck, Darmstadt, Germany).

### Synovial explants

Synovial tissue pieces obtained from an orthopedic hip-replacement surgery in an RA patient were selected based on maximal macroscopic score of inflammation, dissected and seeded on 24-well plates in RPMI, supplemented with 10% heat-inactivated fetal calf serum (FCS), 2 m*M *L-glutamine, 50 IU/ml penicillin, and 50 μg/ml streptomycin (Gibco), and incubated at 37°C with 5% CO_2 _in a humidified atmosphere for 24 hours with 100 μ*M *DXM. Tissue pieces were then transferred to six-well plates and cultured for additional 5 days with fresh prepared serum supplemented with the same concentrations of the drugs. At the end of the incubation, medium was removed, and tissue pieces were cryosectioned, fixed for 20 minutes at 4°C with 2% (vol/vol) formaldehyde (Merck), and stored at -80°C.

### Immunohistochemistry and histologic evaluation

Presence of citrullinated proteins (CPs) was detected by using a mouse IgM monoclonal antibody (F95), which was raised against a decacitrullinated peptide linked to the carrier protein, keyhole limpet hemocyanin [9-11]. For detection of PAD enzyme expression, we used two PAD4 (SN823 and SG1467) [12,13] and one PAD2 (SN665) [12] rabbit polyclonal antibodies and one additional rabbit polyclonal antibody (ROI002) (CosmoBio, Tokyo, Japan) to identify PAD2 expression [14]. Appropriate negative controls were used for each antibody.

Two independent observers (DM and AIC), who were unaware of the sample's identity, scored the presence of CP and PAD enzymes in synovial tissue by using a 4-point scale (0, no staining; 1, low amounts of staining; 2, moderate amounts of staining; 3, high amounts of staining). In parallel, histologic scoring of the degree of inflammation was performed, by using a 4-point scale, by double-blind semiquantitative analysis (DM and SR) of the lining thickness, infiltration level, and vascularity in serial hematoxylin eosin staining. In every instance, the final scores represent the mean of the two observations. The presence of CP and PAD enzymes in SFMCs and PBMCs was assessed with manual counting of positive cells and expressed as a percentage of positive cells in the total number of the cells.

### Statistical analysis

Statistical analysis was performed by using the Wilcoxon test for comparison of paired samples, the Mann-Whitney test for comparison of independent samples, and the Spearmen rank correlation test. All comparisons were planned, and therefore, no Bonferroni correction was applied. Differences between proportions were analyzed with the Fisher Exact test. *In vitro *experiments results were analyzed by using one-way (ANOVA) analysis of variance, as appropriate. *P *values less than 0.05 were considered statistically significant.

## Results

### Intracellular but not extracellular presence of citrullinated peptides is RA specific

**Figure 1:**
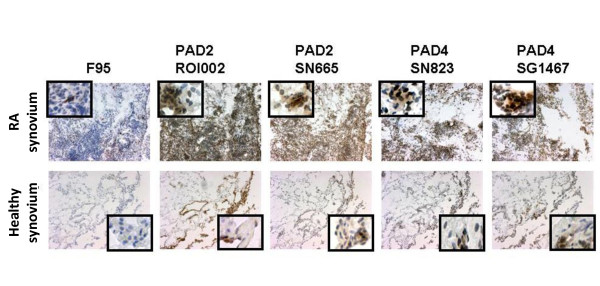
**Representative picture showing that expression of intracellular and total citrullinated protein (CP), as well as PAD2 and PAD4 enzymes, is higher in rheumatoid arthritis (RA; upper panel) as compared with healthy (lower panel) synovial tissue**. Frozen synovial biopsy sections show diaminobenzidine (brown) immunoperoxidase staining (hematoxylin counterstained) for citrullinated proteins and peptidylarginine deiminase (PAD) enzymes detected with the F95 antibody, anti-PAD2 (with two different antibodies, ROI002 and SN665), and PAD4 (with two different antibodies, SN823 and SG1467) antibodies (original magnification, ×100). Insets show close-up details from the same samples.

It was previously suggested that the intracellular pattern of F95 staining is a specific trait of RA as compared with healthy synovium [11]. Further to investigate this, we analyzed the immunohistochemical expression of citrullinated peptides (as detected by F95 antibody) and the PAD2 and PAD4 enzymes in baseline RA samples obtained before initiation of treatment, as compared with healthy synovial biopsies. Intracellular F95 staining was readily detected in RA samples (24 of 28, 85.7%) and absent in healthy synovium (none of eight; *P *< 0.05). Extracellular F95 staining was present in RA (24 of 28, 85.7%) and to a lesser extent in healthy synovial tissue (one of eight, 12.5%; *P *< 0.05). PAD2 and PAD4 expression was detected in all baseline RA samples (100%) and a vast majority of the healthy synovial biopsies (seven of eight, 87.5% positive for ROI002 antibody; six of eight, 75% positive for SN665 antibody; all eight, 100% positive for SG1467 antibody, and seven of eight, 87.5% positive for SN8233 antibody) and with a general higher expression of all investigated molecules in RA as compared with healthy synovium (Figure 1; *P *< 0.05 for all investigated antibodies).

### Synovial citrullination and PAD expression correlate with the degree of inflammation

To investigate further the relation between inflammation and citrullination, we analyzed the correlation between the presence of total and intracellular CP, as well as PAD enzymes, and the degree of local inflammation in baseline samples obtained from all RA patients and healthy individuals (*n *= 34). As expected, most of the investigated markers correlated with the degree of lining thickness, cell infiltration, and vascularity (Table 1).

**Table 1:** Correlations between CP, PAD2, and PAD4 expression and synovial inflammation

	Mean lining thickness	Infiltration	Vascularity
Total CP	0.4 (< 0.05)	0.5 (< 0.001)	0.6 (< 0.001)
Intracellular CP	0.4 (< 0.05)	0.4 (0.001)	0.6 (< 0.01)
PAD2 (ROI002)	0.5 (< 0.05)	0.6 (< 0.001)	0.4 (< 0.05)
PAD2 (SN665)	0.6 (< 0.001)	0.7 (< 0.001)	0.5 (< 0.01)
PAD4 (SG1467)	0.6 (< 0.01)	0.3 (NS)	0.3 (NS)
PAD4 (SN823)	0.5 (< 0.01)	0.1 (NS)	0.2 (NS)

Values are expressed as Spearman rank correlation coefficients (*P *values). CP, citrullinated protein; NS, not significant.

### Intraarticular GC, but not MTX, decreases synovial citrullination and PAD4 expression

The observed correlation between inflammation and citrullination prompted us to investigate the effect of antirheumatic treatment on synovial citrullination and PAD expression.

**Figure 2:**
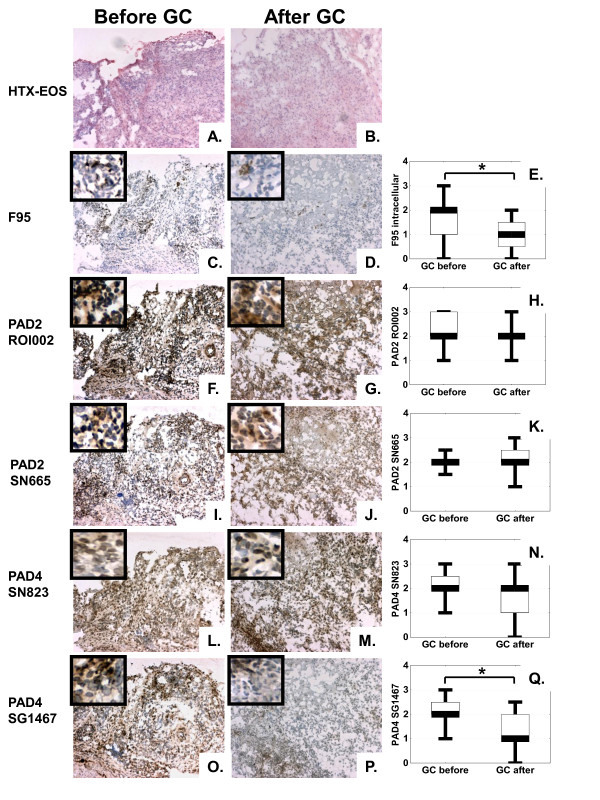
**Intraarticular glucocorticoid (GC) treatment of rheumatoid arthritis (RA) patients (*n *= 15) decreases synovial expression of F95 and peptidylarginine deiminase 4 (PAD4) but not PAD2**. Hematoxylin-eosin (HTX-EOS) staining illustrates the histologic pattern of frozen synovial biopsy sections of an RA patient before **(a) **and after **(b) **intraarticular GC treatment. Brown diaminobenzidine immunoperoxidase staining shows a significant decrease in the expression of citrullinated proteins, as detected by F95 antibody (**c**, before treatment; **d**, after treatment), no changes in PAD2 expression as detected by either ROI001 (**f**, before treatment; **g**, after treatment) antibody or SN665 antibody (**i**, before treatment; **j**, after treatment) and a significant decrease in PAD4 expression, as detected by SG1467 antibody (**o**, before treatment; **p**, after treatment) but not by SN823 antibody (**l**, before treatment; **m**, after treatment). Original magnification, ×100; insets show close-up details from the same samples. Graphs show results of semiquantitative analysis on a 4-grade scale for citrullinated proteins **(e)**, PAD2 as detected with either ROI001 **(h) **or SN665 **(k) **antibody, and PAD4 as detected with either SN823 **(n) **or SG1467 **(q) **antibody. Values represent the median, and whiskers, the range (*P < 0.05).

Local administration of GC significantly decreased both total and intracellular expression of CP from a median score of 2 (range, 0 to 3) to a median score of 1 (range, 0 to 2), *P *< 0.05. PAD4 expression also decreased after treatment from a median score of 2 (range, 1 to 3) to a median score of 1 (range, 0 to 2.5), *P *< 0.05, when evaluated with the SG1467 antibody and to a lesser extent when evaluated with the SN823 antibody (*P *= 0.06), whereas no changes were observed for PAD2 expression after intraarticular administration of GCs (Figure 2). The GC effect on citrullination and PAD expression was paralleled by a significant decrease (*P *< 0.05) in the lining thickness (from a median score of 1, range, 0 to 2, to a median score of 0.5, range, 0 to 1) and cell infiltration (from a median score of 3, range, 2 to 3, to a median score of 2, range, 1 to 3), but not vascularity. All patients were good clinical responders, as evaluated with macroscopic investigation of the joint inflammation at the time of arthroscopy and retrospective scoring of photo records.

**Figure 3:**
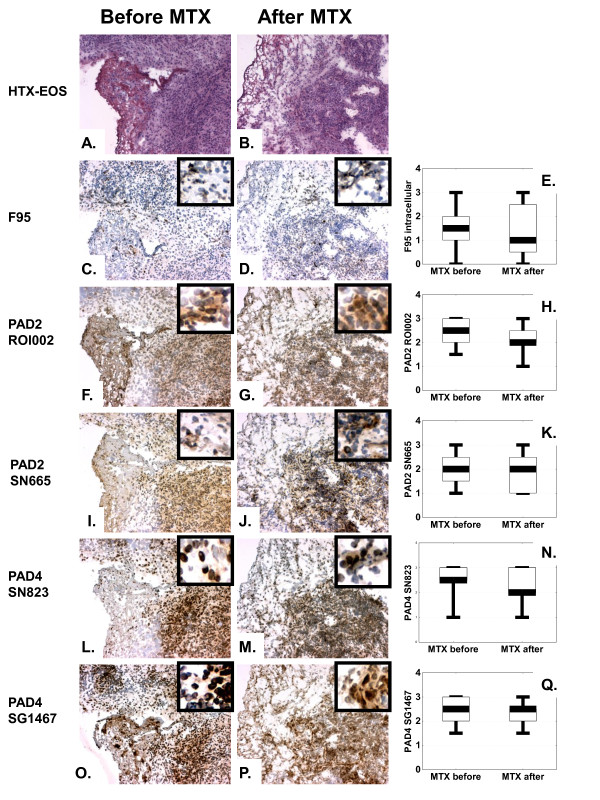
**Methotrexate (MTX) treatment of rheumatoid arthritis (RA) patients (*n *= 11) does not modulate synovial expression of either citrullinated proteins or PAD2 and PAD4 enzymes**. Hematoxylin-eosin (HTX-EOS) staining illustrates the histologic pattern of frozen synovial biopsy sections of an RA patient before **(a) **and after **(b) **MTX treatment. Brown diaminobenzidine immunoperoxidase staining shows no changes in expression of citrullinated proteins as detected by F95 antibody (**c**, before treatment; **d**, after treatment), PAD2 as detected with either ROI001 antibody (**f**, before treatment; **g**, after treatment), or SN665 antibody (**i**, before treatment; **j**, after treatment) and PAD4 as detected with either SN823 antibody (**l**, before treatment; **m**, after treatment), or SG1467 antibody (**o**, before treatment; **p**, after treatment). Original magnification ×100, whereas insets show close-up details from the same samples. Graphs show results of semiquantitative analysis on a 4-grade scale for citrullinated proteins **(e)**, PAD2 as detected with either ROI001 **(h) **or SN665 **(k) **antibody, and PAD4 as detected with either SN823 **(n) **or SG1467 **(q) **antibody. Values represent the median, and whiskers, the range (*P < 0.05).

In contrast, methotrexate treatment had no effect on either synovial inflammation or local expression of CP and PAD (Figure 3), despite a good clinical response in nine of 11 treated patients with a significant decrease in the DAS28 score from a mean ± SEM of 5.5 ± 0.3 to a mean ± SEM of 3.4 ± 0.4 after 3 months of treatment. Interestingly, the same was true when only EULAR responders (*n *= 9) were included in the analysis.

### *In vitro *GCs have a direct effect on cellular expression of CP, potentially through a PAD-dependent mechanism

**Figure 4:**
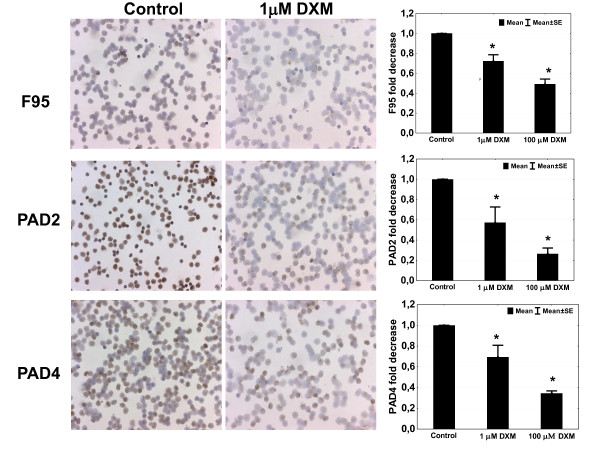
**Dexamethasone (DXM) decreases the expression of citrullinated proteins and PAD4 and PAD2 in synovial fluid mononuclear cells (SFMCs), in a dose-dependent manner**. Brown diaminobenzidine immunoperoxidase staining detects citrullinated proteins, as detected with F95 antibody, PAD2 as detected with ROI001 antibody, and PAD4 as detected with SN823 antibody. Original magnification, ×100. Graphs show results of manual counting in six different SFMC samples analyzed in duplicate, and results are expressed as fold decrease of the number of positive cells in the treated samples as compared with controls.

**Figure 5:**
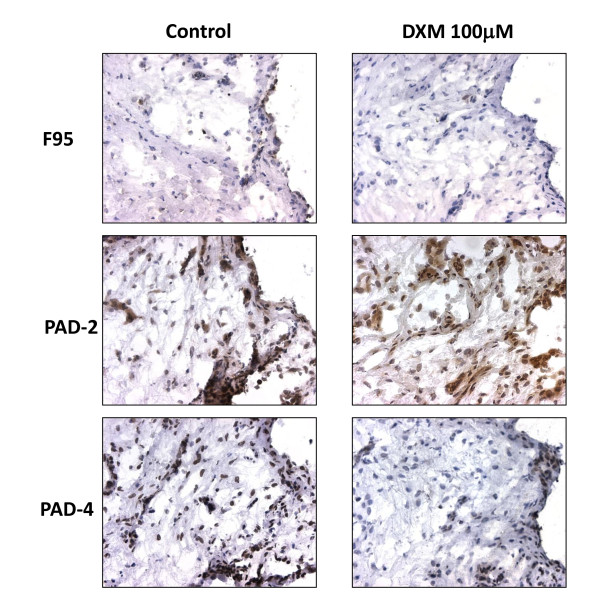
**Dexamethasone (DXM) decreases the expression of citrullinated proteins and PAD4 expression in a rheumatoid arthritis (RA) synovial explant**. Brown diaminobenzidine immunoperoxidase staining shows citrullinated proteins as detected with F95 antibody, PAD2 as detected with ROI001 antibody, and PAD4 as detected with SN823 antibody. Original magnification, ×250.

Apart from their inflammation-damping effect, GCs may also directly target the process of citrullination. To investigate this, we tested the effect of DXM on CP and PAD enzymes expression *in vitro *in SFMC and PBMC paired samples of RA individuals. Confirming our *in vivo *results, DXM decreased the expression of CP, PAD4, but also PAD2, in SFMC (Figure 4) but not PBMC (data not shown). The effect of DXM is dose dependent and present at doses as low as 1 μ*M *(Figure 5). No such effects were observed for methotrexate when tested in the same systems (Additional file 1).

### GC effect can be reproduced *ex vivo *in synovial explants biopsies

SFMCs are *in vitro *surrogate replacements of the synovial biopsy complex milieu, although lacking important characteristics, such as the local complex intracellular interplay. To circumvent this caveat, we established explants from a synovial biopsy obtained from an RA patient at open surgery for hip replacement and tested the effect of GCs to confirm our results further. DXM treatment of the explants decreased the expression of CP and PAD4 but not PAD2, whereas parallel histologic investigation did not reveal major changes in the histologic composition of the samples. Once again, no such effects were observed for methotrexate (Additional file 2).

## Discussion

Protein modification through posttranslational citrullination in the rheumatoid joint is thought to play an important role in perpetuation of local chronic inflammation in the presence of specific anticitrulline immunity. This is the first report showing that synovial citrullination is actively modulated by antirheumatic treatment.

Previous studies characterized synovial expression of citrullination, showing a general increase in the level of citrullination in the presence of active inflammation [2,4-6,11,15]. These early reports used one particular method for detection of citrullination, consisting of the chemical modification of the citrullinated tissue proteins to allow their recognition by an antibody raised against similarly modified citrullinated proteins [16]. However, this technique is no longer available, and in our hands, the only other antibody performing well in immunohistochemistry is F95 [10]. As with the modified anti-citrulline antibody, F95 is supposed to recognize a large array of citrullinated molecules independent of the amino acid context. We previously demonstrated that this antibody is able to recognize specifically at least one RA-relevant citrullinated antigen, citrullinated fibrinogen [17]. However, a somewhat more restricted pattern of citrullination, as compared with the modified anti-citrulline antibodies, has been observed both in synovial biopsies [11] and in other tissues, such as lungs of smoking healthy individuals [18]. By using this new antibody, De Rycke *et al. *[11] were able to describe an RA-specific intracellular F95 staining. We confirm this finding by the virtually absence of F95-positive cells in synovial biopsies of healthy individuals.

Only few reports of PAD synovial expression are currently available [2,11,19]. The most thorough investigation of synovial PAD expression was performed by Foulquier *et al. *[2], showing correlation between expression of both PAD2 and PAD4 expression with the degree of synovial inflammation. We confirm these findings and identify the same correlation for the CP expression.

The novel finding of the current study is the reduction in citrullination and PAD expression induced by intraarticular GC, which was not observed after MTX treatment. One potential explanation for this difference is the different time points to obtain the follow-up biopsy, 2 weeks after treatment initiation with GCs, as compared with 8 weeks for MTX. However, the two different time points were chosen in accordance with the clinical expectation of maximal effect of the administered drug, which would theoretically increase the chance to observe a change, not only for GCs, but also for MTX. We were able also to demonstrate a direct effect of GCs independent of inflammation in our synovial explants. This is further supported by the results of the *in vitro *experiments, in which we observed a dose-dependent effect of GCs despite obvious difficulties in standardization of a quantitative analysis by using immunohistochemistry on cells. Interestingly, although GCs decreased citrullination and PAD4 expression in SFMCs, no such effect was observed in PBMCs. This could be due to the lower baseline levels and, as a consequence, to the lower sensitivity to detect changes of expression of the investigated molecules in PBMCs as compared with SFMCs. Conversely, it could be due to different regulatory mechanisms and cellular activation states of PBMCs, as compared with SFMCs, as previously suggested [20]. Despite major advances in understanding the central role of citrullination and anti-citrulline immunity in RA pathogenesis, we face a striking lack of knowledge regarding regulatory factors responsible for induction, perpetuation, and/or amelioration of the process of citrullination, both locally in the joint and even more generally in other tissues where citrullination occurs either under physiologic (skin) [21] or pathologic conditions (lungs of smokers) [18,22]. It is generally accepted that citrullination accompanies inflammation, and it has been suggested that induction of citrullination by inflammatory stimuli such as TNF occurs after PAD4 activation and induction of signaling pathways dependent on NF-κB [23]. Interestingly, the antiinflammatory effects of GCs are at least partially dependent on NF-κB, as demonstrated by their lack of effect in animal models of acute inflammation in NF-κB knockout mice [24]. In contrast, MTX appears to have limited effects on NF-κB activation, as demonstrated by high levels of activation in PBMCs of MTX-treated RA patients that can be reversed by anti-TNF agents [25]. These findings suggest that GCs might affect citrullination through PAD4 downregulation through an NF-κB-dependent mechanism.

## Conclusion

The inflamed RA synovium is characterized by high expression of intracellular CP and PAD4 enzyme that is reversed through GCs, but not MTX treatment. Further investigation of the exact mechanism of action and comparison with other antirheumatic drugs is warranted.

## Abbreviations

ACPA: anti-citrullinated protein antibodies; CP: citrullinated protein; DMARD: disease-modifying antirheumatic drug; DXM: dexamethasone; FCS: fetal calf serum; GC: glucocorticoid; MC: mononuclear cell; MTX: methotrexate; PAD: peptidylarginine deiminase; PB: peripheral blood; RA: rheumatoid arthritis; SF: synovial fluid.

## Competing interests

The authors declare that they have no competing interests.

## Authors' contributions

DM, AIC, and SR participated in study design, collection and interpretation of the data, and manuscript writing. ME and EaK participated in the collection of data. APN and GP participated in data collection, interpretation of the data, and manuscript writing. DM and SR contributed equally to this work. All authors read approved the final manuscript.

## Supplementary Material

Additional file 1 **Methotrexate (MTX) has no effect on expression of citrullinated proteins and PAD4 and PAD2 in SFMCs**. Brown diaminobenzidine immunoperoxidase staining detects citrullinated proteins, as detected with F95 antibody, PAD2 as detected with ROI001 antibody, and PAD4 as detected with SN823 antibody. Original magnification, ×200. Graphs show results of manual counting in six different SFMC samples analyzed in duplicate, and results are expressed as fold decrease of the number of positive cells in the treated samples as compared with controls. Click here for file

Additional file 2 **MTX has no effect on expression of citrullinated proteins and PAD4 expression in an RA synovial explant**. Brown diaminobenzidine immunoperoxidase staining shows citrullinated proteins as detected with F95 antibody, PAD2 as detected with ROI001 antibody, and PAD4 as detected with SN823 antibody. Original magnification, ×250. Click here for file
