# Supplementary figures and images for: Local administration of glucocorticoids decreases synovial citrullination in rheumatoid arthritis
Source: Arthritis Res Ther. 2012 Jan 27;14(1):R20. doi: 10.1186/ar3702 (PMC3392813; doi:10.1186/ar3702)

## Slide 1
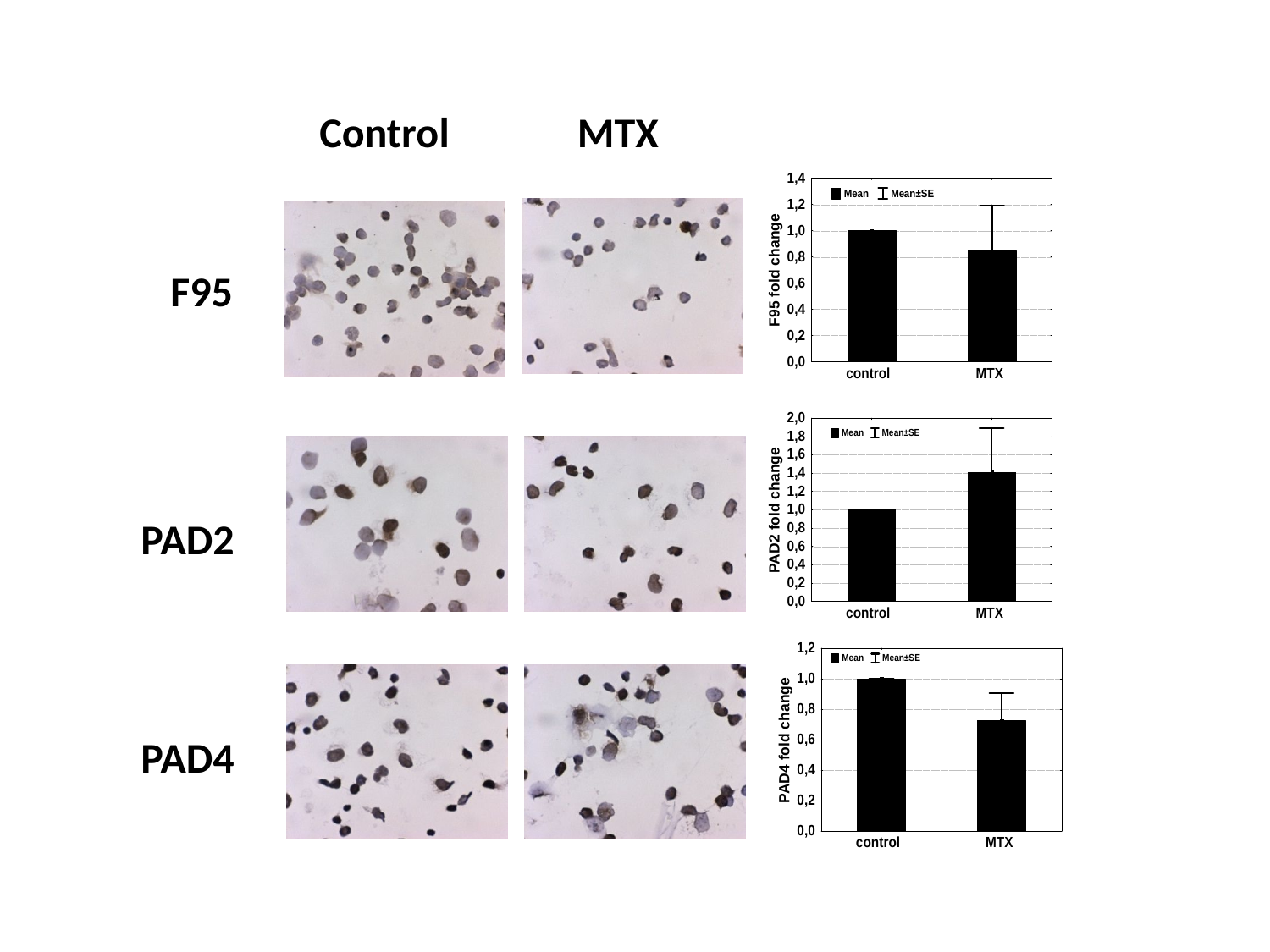

Control
MTX
F95
PAD2
PAD4

Supplement: Additional file 1 — Methotrexate (MTX) has no effect on expression of citrullinated proteins and PAD4 and PAD2 in SFMCs. Brown diaminobenzidine immunoperoxidase staining detects citrullinated proteins, as detected with F95 antibody, PAD2 as detected with ROI001 antibody, and PAD4 as detected with SN823 antibody. Original magnification, ×200. Graphs show results of manual counting in six different SFMC samples analyzed in duplicate, and results are expressed as fold decrease of the number of positive cells in the treated samples as compared with controls. [file ar3702-S1.PPT]

## Slide 1
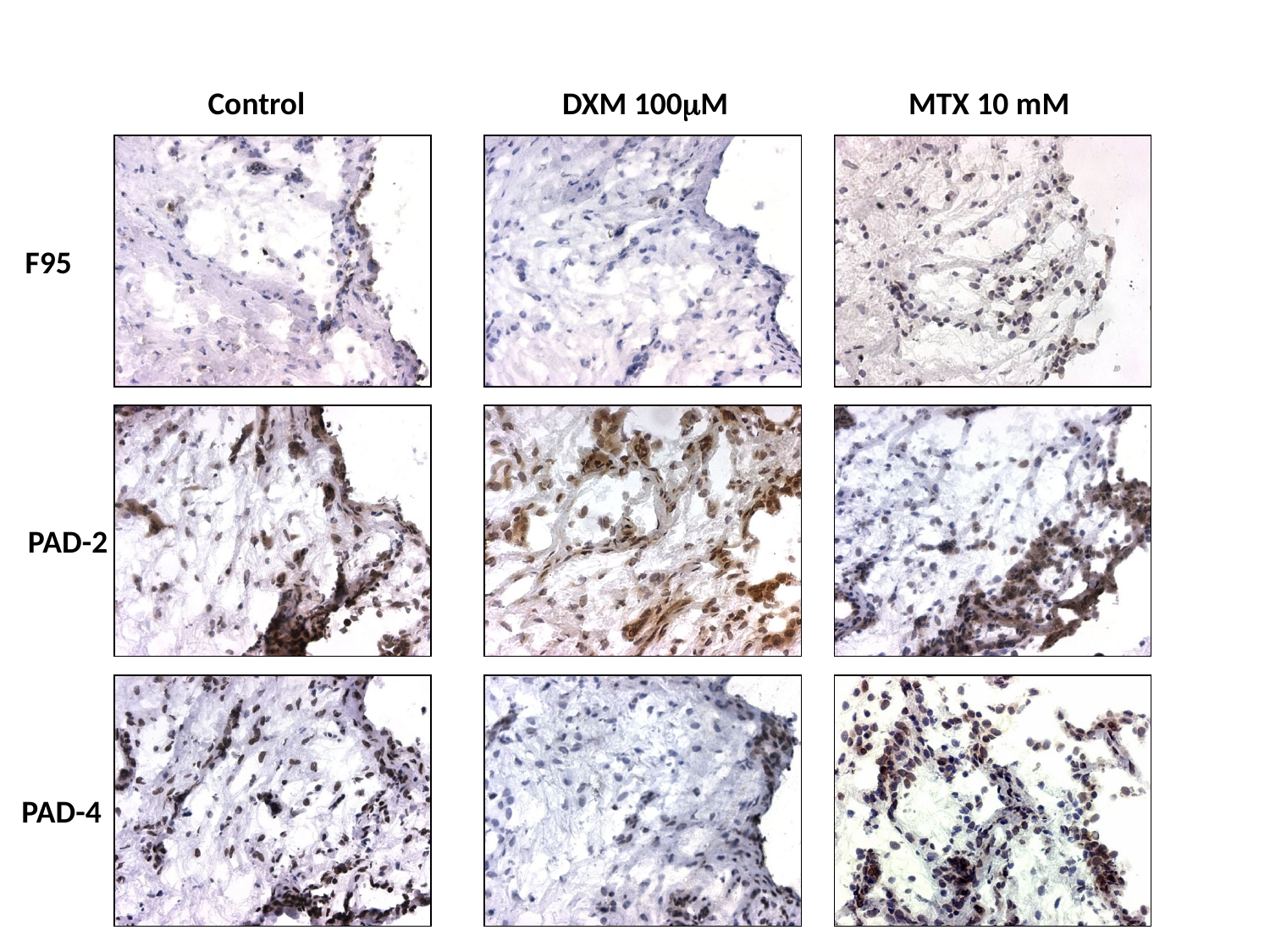

Control
DXM 100μM
MTX 10 mM
F95
PAD-2
PAD-4

Supplement: Additional file 2 — MTX has no effect on expression of citrullinated proteins and PAD4 expression in an RA synovial explant. Brown diaminobenzidine immunoperoxidase staining shows citrullinated proteins as detected with F95 antibody, PAD2 as detected with ROI001 antibody, and PAD4 as detected with SN823 antibody. Original magnification, ×250. [file ar3702-S2.PPT]
